# A Generalized Lossy Transmission-Line Model for Tunable Graphene-Based Transmission Lines with Attenuation Phenomenon

**DOI:** 10.1038/srep31760

**Published:** 2016-08-24

**Authors:** Yongle Wu, Meijun Qu, Yuanan Liu

**Affiliations:** 1 Beijing Key Laboratory of Work Safety Intelligent Monitoring, School of Electronic Engineering, Beijing University of Posts and Telecommunications, P.O. Box. 282, 100876, Beijing, China

## Abstract

To investigate the frequency shift phenomenon by inserting graphene, a generalized lossy transmission-line model and the related electrical parameter-extraction theory are proposed in this paper. Three kinds of graphene-based transmission lines with attenuation phenomenon including microstrip line, double-side parallel strip line, and uniplanar coplanar waveguide are analyzed under the common conditions where different chemical potentials are loaded on graphene. The values of attenuation constant and phase constant, and the real and imaginary parts of the characteristic impedance of transmission lines are extracted to analyze in details. When the attenuation constant and the reactance part of the characteristic impedance are approximately equal to zero, this kind of transmission line has low or zero insertion loss. On the contrary, the transmission line is under the radiation mode with obvious insertion loss. The phase constant changes linearly under the transmission mode and can be varied with changing of chemical potentials which attributes to the property of frequency tunability. Furthermore, a bandwidth reconfigurable uniplanar coplanar waveguide power divider is simulated to demonstrate that this theory can be applied to the design of three-port devices. In summary, this work provides a strong potential approach and design theory to help design other kinds of terahertz and mid-infrared reconfigurable devices.

Graphene is a promising candidate for nanoelectronics and nanophotonics devices due to its remarkable electro-optical property^1^,^2^. Graphene has aroused great interest and its potential applications for various fields have been explored recently^3^,^4^,^5^,^6^,^7^,^8^,^9^,^10^,^11^. For example, the reported graphene-based devices include absorbers^3^, electro-optical switches^4^, field-effect transistors^5^, amplifiers^6^, diodes^7^, terahertz antennas^8^, filters^9^, mixers^10^, plasmonic bragg reflectors^11^, and so on. The richness of optical and electronic properties makes the graphene have multiple attractive features. A multimode interferometer^12^ in mid-infrared spectrum based on a dielectric-loaded graphene plasmonic waveguide has been designed. This waveguide has extremely large modal effective indexes and modal effective index contrast, which can shrink the multimode interferometer greatly. The transparent graphene is considered as a front electrode in Organic/GaAs hybrid photovoltaic cells^13^, and the power conversion efficiency can be greatly enhanced. In addition, the graphene is applied in a copper-graphene-based photonic crystal fiber plasmonic biosensor^14^ to prevent Cu oxidation and enhance sensing performance.

The surface complex conductivity of the graphene can be dynamically controlled by the applied voltage, which makes it possess unprecedented opportunities for reconfigurable plasmonic devices at terahertz and mid-infrared frequencies. The operating bandwidth of the broadband absorber based on graphene can be dynamically tuned by varying the bias voltage of the graphene^3^. A novel reconfigurable terahertz graphene-exploited leaky-wave antenna can allow both frequency tuning and beam steering by adjusting the graphene conductivity^8^. The design of the tunable optical delay line is continuously tunable due to the graphene sandwiched between the stacked ring resonators^15^. Tuning of the delay time could be achieved by varying the voltage across the graphene layers.

However, few researches have investigated the fundamental theory for explaining the frequency shift characteristic of graphene-loaded devices and providing the design guides. To our best knowledge, the generalized lossy transmission-line model and electrical parameter-extraction (T-LMEP-E) theory are proposed for the first time in this paper to design frequency tunable graphene-based transmission lines. Three kinds of practical and common transmission lines including microstrip, double-side parallel strip line and uniplanar coplanar waveguide are chosen and investigated. The calculated results based on the proposed T-LMEP-E theory show that the introduction of graphene in transmission line greatly affects the phase constant modeled as a function of frequency. In the linear changing range of the phase constant at a certain frequency point, different chemical potentials loaded in graphene correspond to different phase constant. Thus the changing of chemical potential of the graphene could alter the phase constant, so as to vary the resonant frequency of the transmission line. When the attenuation and the reactance part of the characteristic-impedance approximately equal to zero, the transmission line has low or zero insertion loss. Otherwise, the transmission line exhibits a large insertion loss under the radiation mode. In order to verify the application of the proposed T-LMEP-E theory on the three-port devices, a bandwidth reconfigurable uniplanar coplanar waveguide power divider is simulated. Finally, these results show that the proposed T-LMEP-E theory would provide an effective and clear design guide for reconfigurable terahertz and mid-infrared graphene-based devices.

## Methods

### Lossy Transmission-Line Model and Electrical Parameter-Extraction (T-LMEP-E) Theory

**Figure 1:**
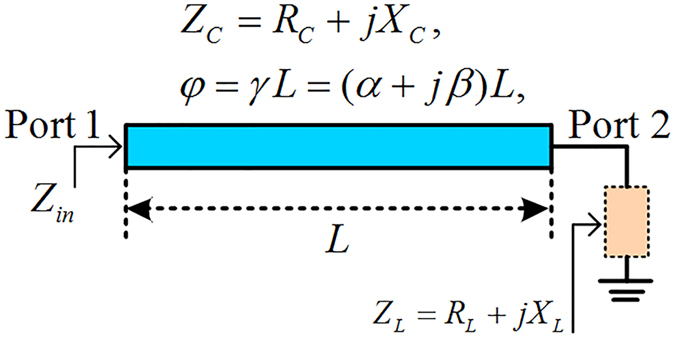
The lossy transmision-line model with defined parameters.

In this work, we propose the generalized transmission-line concept and the corresponding mathematical model for accurately characterizing the two-port network parameters of the microstrip line, the double-side parallel strip line, and the uniplanar coplanar waveguide. The proposed transmission-line concept and model especially for lossy feature are very convenient and effective to predicate the attenuation phenomenon of three kinds of transmission lines. Unlike the lossless transmission-line theory, which is widely applied in microwave engineering^16^, the lossy transmission-line model requires complex propagation constant and complex characteristic impedance in its mathematical expressions. As shown in Fig. 1, the complex propagation constant *γ* = (*α* *+* *jβ*) is defined, where *α* and *β* are the attenuation and phase constants, respectively. Similarly, the complex characteristic impedance is defined as *Z*_*c*_ *=* *R*_*c*_ *+* *jX*_*c*_, where *R*_*c*_ and *X*_*c*_ are the real and imaginary parts, respectively. If the physical length of the transmission line is *L* and the terminated impedance is *Z*_*L*_, the input impedance *Z*_*in*_ can be calculated by


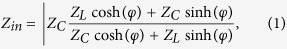


where


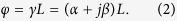


In order to achieve the analytical and frequency-dependent scattering parameters, the ABCD matrix of this proposed transmission-line model shown in Fig. 1 can be expressed by


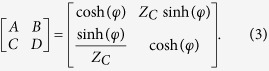


When the port impedances of both Ports 1 and 2 are equal to 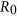, the ABCD matrix of this proposed transmission-line model can be determined by the final scattering parameters (for both the simulation and the measurement results)^17^. The mathematical expressions are


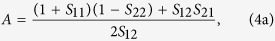



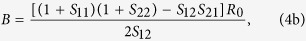



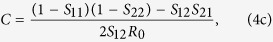



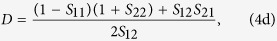


where *S*_*ij*(=1,2)_ can be arbitrary simulated or measured results. After combing (1–4), we can obtain the following equations to calculate the complex propagation constant and the characteristic impedance:


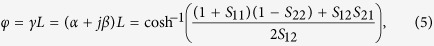



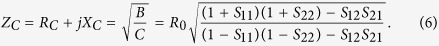


Since the scattering parameters *S*_*ij*( = 1,2)_ are the functions of the operating frequency *f*, namely, frequency-dependent feature, the complex propagation constant γ and the characteristic impedance *Z*_*c*_ are also varied with the operating frequency *f*. The graphene can be modeled as a two-dimensional surface with complex conductivity σ due to its property of an atom thickness^18^. The intra-band and inter-band conductivities defined by Kubo’s formulas are given as^19^,


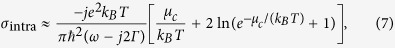



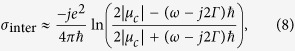


where *e* is the electron charge, *ω* is the angular frequency, *k*_*B*_ is the Boltzmann constant, 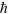 is the reduced Plank constant, *T* is the temperature in Kelvin, *τ* is the electron relaxation time, and *Г* = 1/(2*τ*) is the electron scattering rate. In this paper, the common condition that *T* = 300 K and *τ* = 1 ps is assumed^20^, and only the intra-band contribution is considered due to the slight influence of the inter-band conductivity. It should be noted that the technology of the hybrid graphene-metal implementation has been validated in the experiment^21^.

### Numerical calculations

All models in this paper are simulated using the full-wave electromagnetic simulator software based on the finite element method (FEM). The determined substrate is constructed by using SiO_2_ with a dielectric constant of 3.8 and a thickness of 1 *μ*m. The hybrid graphene-metal implementation is a novel method^21^,^22^,^23^,^24^ to make the whole transmission line have dynamic equivalent surface conductivity, which can be tuned by changing the chemical potential.

## Results

### Three kinds of transmission lines and the graphene-based power divider using uniplanar coplanar waveguide transmission line

**Figure 2:**
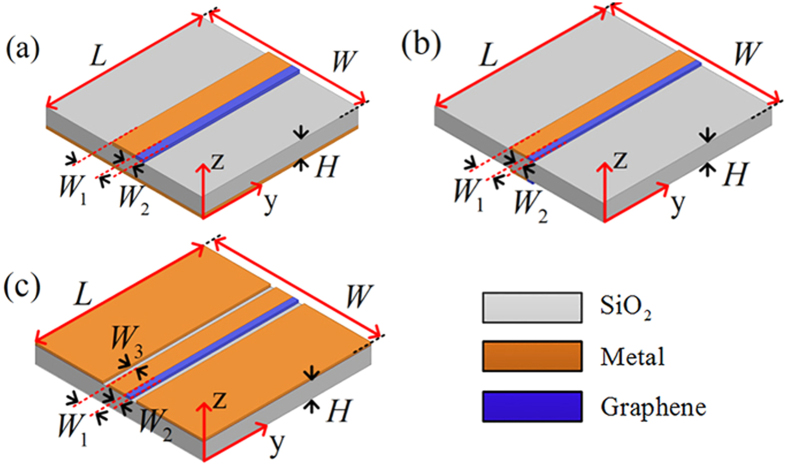
Three-dimensional view of the three kinds of transmission lines. (a) Configuration of the graphene-metal microstrip transmission line. The top layer of this microstrip line includes two adjacent parts (metal and graphene), and the graphene is shown as blue part. The bottom complete metal layer can be regarded as a ground for microstrip line. The physical parameters are set as *W* = 30 *μ*m, *W*_1_ = 2 *μ*m, *W*_2_ = 0.5 *μ*m, *L* = 60 *μ*m, and *H* = 1 *μ*m. (b) Schematic diagram of the graphene-based double-side parallel strip transmission line. The top and bottom layers are identical while the middle of the substrate is equivalent to a virtual ground. The parameters are chosen as *W* = 30 *μ*m, *W*_1_ = 2.4 *μ*m, *W*_2_ = 0.5 *μ*m, *L* = 60 *μ*m, and *H* = 1 *μ*m. (c) The 3D geometry of the graphene-based uniplanar coplanar waveguide transmission line. It is the most suitable transmission line in mid-infrared and terahertz frequencies due to the signal line and ground are on the same layer. The values of geometrical parameters are *W* = 30 *μ*m, *W*_1_ = 1.7 *μ*m, *W*_2_ = 0.5 *μ*m, *W*_3_ = 1 *μ*m, *L* = 60 *μ*m, and *H* = 1 *μ*m.

**Figure 3:**
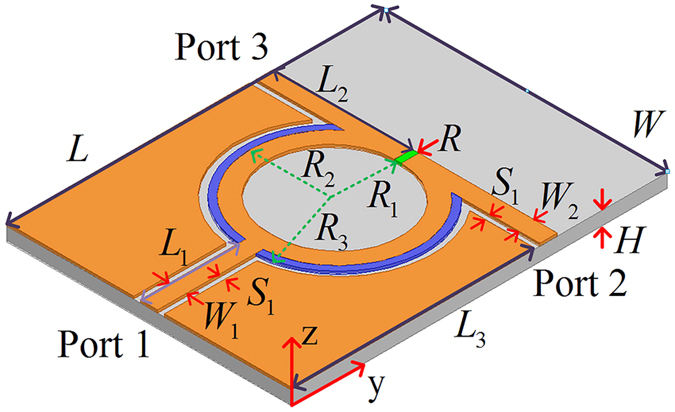
The schematic of the graphene-based power divider adopting uniplanar coplanar waveguide (strip) transmission line. The power divider is all mounted on a substrate with the same form as the uniplanar coplanar waveguide (strip) transmission line. Port 1 is the input port of the power divider, while the port 2 and port 3 are output ports, respectively. The isolation resistor *R* is added in power divider to achieve good isolation between two output ports. The optimized parameters are *W* = 42.8 *μ*m, *W*_1_ = 3.1 *μ*m, *W*_2_ = 2.6 *μ*m, *L* = 45.9 *μ*m, *L*_1_ = 15.1 *μ*m, *L*_2_ = 20.6 *μ*m, *L*_3_ = 32.5 *μ*m, *R*_1_ *=* 7.4 *μ*m, *R*_2_ *=* 10 *μ*m, *R*_3_ *=* 10.5 *μ*m, *S*_1_ *=* 0.5 *μ*m, *H* = 1 *μ*m, and *R* = 50 ohm.

The three kinds of transmission lines are analyzed by using the generalized lossy transmission-line concept and the corresponding mathematical model. The general propagation constant *γ* = (*α* *+* *jβ*) and the characteristic impedance *Z*_*c*_ *=* *R*_*c*_ *+* *jX*_*c*_ are both complex values, where *α* and *β* are the attenuation and phase constants while *R*_*c*_ and *X*_*c*_ are the real and imaginary parts of the characteristic impedance, respectively. The parameters of *α*, *β*, *R*_*c*_, and *X*_*c*_ of the three kinds of transmission lines are extracted to model as a function of frequency. The scattering parameters of the three kinds of transmission lines and the uniplanar coplanar waveguide (strip) power divider are simulated by the full-wave electromagnetic simulation software. Three-dimensional views of the graphene-based microstrip, the double-side parallel strip and the uniplanar coplanar waveguide transmission lines are presented in Fig. 2 while the uniplanar coplanar waveguide (strip) power divider based on graphene is shown in Fig. 3.

**Figure 4:**
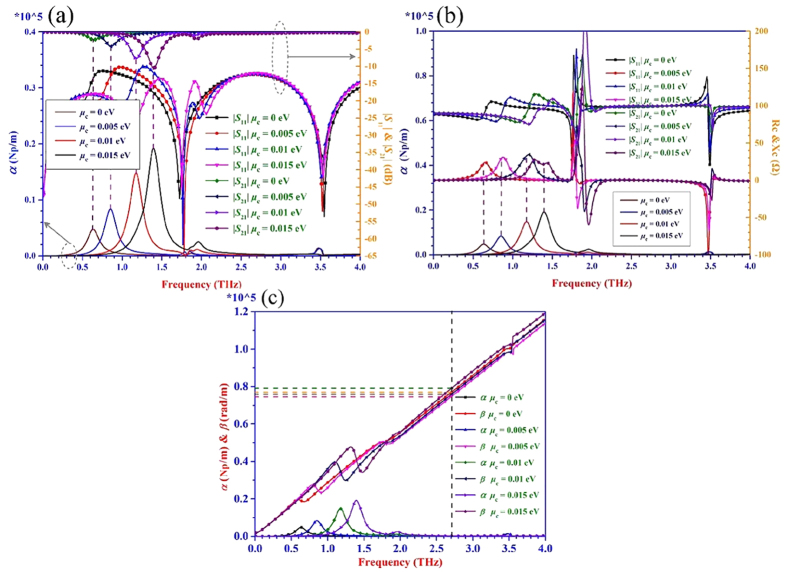
The curves of scattering parameters, *α*, *β*, *R*_*c*_, and *X*_*c*_ of the graphene-metal microstrip line with the varied chemical potential *μ*_c_. (**a**) The |*S*_11_| and |*S*_21_| with the changing of chemical potential. (**b**) The curves of *R*_*c*_ and *X*_*c*_extracted from the simulated scattering parameters when the graphene is loaded with different chemical potentials from 0 eV to 0.015 eV. (**c**) The curves of *α* and *β* obtained from T-LMEP-E theory by altering chemical potential *μ*_c_. The value of *α* is also presented in (**a**,**b**) for the purpose of comparison.

**Figure 5:**
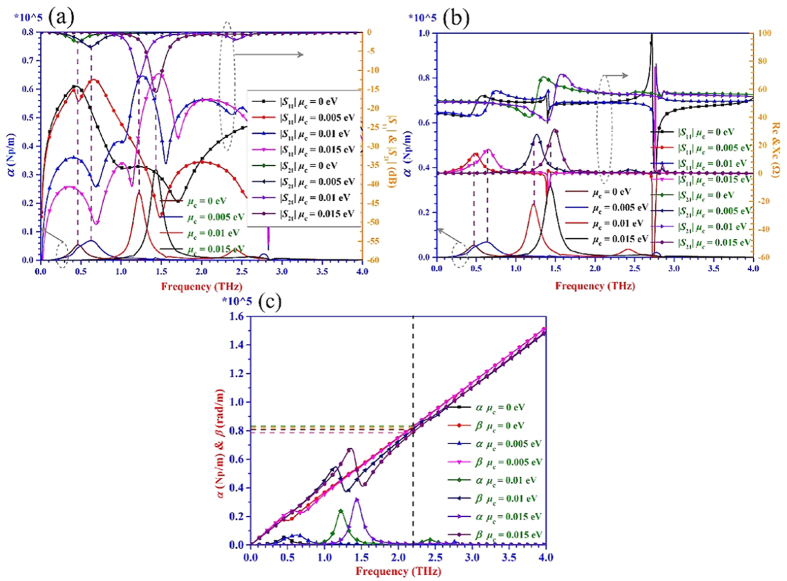
The curves of scattering parameters, *α*, *β*, *R*_*c*_, and *X*_*c*_ of the double-side parallel strip transmission line with the changing of chemical potential based on graphene. (**a**) The curves of |*S*_11_| and |*S*_21_| with the altering of chemical potential. (**b**) The curves of *R*_*c*_ and *X*_*c*_extracted from T-LMEPE theory with varying chemical potentials. (**c**) The calculated results of *α* and *β* from T-LMEP-E theory are displayed when the chemical potential is changing. The curve of *α* is also presented in (**a**,**b**) to make the comparisons more intuitive.

**Figure 6:**
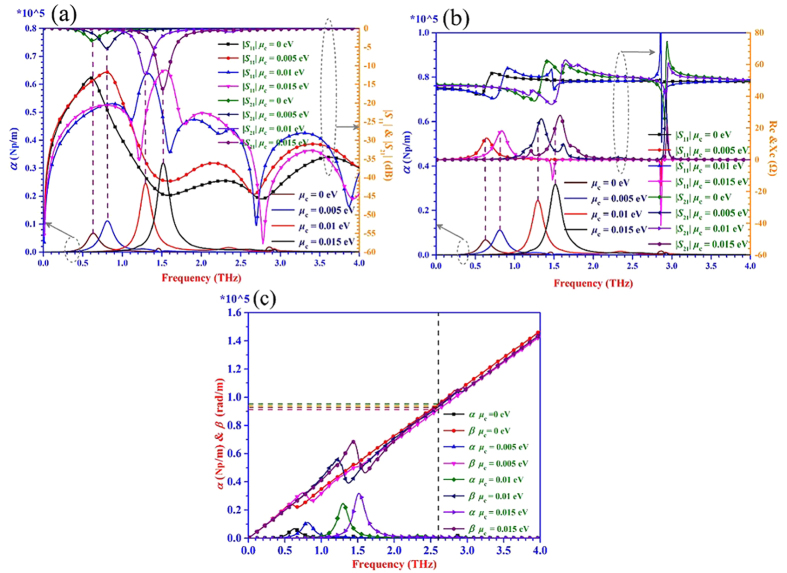
The results of scattering parameters, *α*, *β*, *R*_*c*_, and *X*_*c*_ of the graphene-based uniplanar coplanar waveguide transmission line when the graphene is loaded with different chemical potentials. (**a**) The curves of |*S*_11_| and |*S*_21_| when the chemical potential changed from 0 eV to 0.015 eV. (**b**) The curves of *R*_*c*_ and *X*_*c*_ extracted from T-LMEP-E theory with altering chemical potentials. (**c**) The values of *α* and *β* calculated from the simulated scattering parameters with the dynamic chemical potentials. The value of *α* is also shown in (**a**,**b**) to clarify our proposed theory easily.

The simulated results for the three kinds of transmission lines are shown in Figs 4, 5, 6. It can be seen from Figs 4(a), 5(a) and 6(a) that when the value of |*S*_21_| is almost equal to zero, the attenuation *α* is close to zero. While the value of |*S*_21_| is obviously not equal to zero, the attenuation *α* is not zero as well. The energy can be transmitted from the input port to the output port of the transmission line with a very low loss within the frequency range where the |*S*_21_| is nearly equal to 0 dB. Thus, the transmission lines are under the transmission mode in this situation. Once the |*S*_21_| is not zero and the attenuation *α* has a large value, the energy will be dissipated in the transmission line to a great degree. In this case, the transmission lines are not under the transmission mode but the radiation mode. From Figs 4(a), 5(a) and 6(a), it also can be seen clearly that the frequency is tunable with different chemical potentials loaded on the graphene under the transmission mode. As the chemical potential changes from 0 eV to 0.015 eV, the frequency where meets the condition initially of |*S*_21_| = −3 dB and |*S*_11_| < −10 dB increases.

From the proposed T-LMEP-E theory, the complex characteristic impedance *Z*_*c*_ *=* *R*_*c*_ *+* *jX*_*c*_ is extracted and plotted in Figs 4(b), 5(b) and 6(b) for all the three kinds of transmission lines. When the values of *R*_*c*_ and *X*_*c*_ are in flat area which means the values of them almost maintain the same with the changing of frequencies, the attenuation *α* is close to 0. At the same time, the impedance *R*_*c*_ is about 100 Ω, 55 Ω and 50 Ω extracted from microstrip transmission line, double-side parallel strip transmission line and uniplanar coplanar waveguide transmission line, respectively. And all of the reactance *X*_*c*_ are approximately equal to zero in the three situations, which means the transmission line is under the transmission mode. It would become easy to achieve acceptable impedance transformation and good power transmission characteristics for matching circuits/systems because the characteristic impedance is real. When the *R*_*c*_ and *X*_*c*_ have variable values, the corresponding attenuation *α* has a considerable value. The transmission line is under the radiation mode and the energy is easily dissipated because the reactance has considerable value. The values of the reactance *X*_*c*_ are related to impedance matching which has an influence on transmission characteristics. Thus the proposed T-LMEP-E theory can be used to extract the parameters of attenuation constant and phase constant, the real and imaginary parts of the characteristic impedance and guide the design of graphene-based devices.

The Figs 4(c), 5(c) and 6(c) show that the calculated attenuation constant *α* and phase constant *β* versus the frequency extracted from the proposed lossy transmission-line model and its related theory. Under the transmission mode, the phase constants almost have a linear relationship with frequency. The values of phase constant (*β* = 2π/λ_g_, where λ_g_ is the equivalent wavelength) are different when the chemical potential is altered. Under the transmission mode, the equivalent wavelengths λ_g_ (λ_g_ = *v*_g_/*f*_g_) are unequal when the phase constants are not the same. Therefore, it finally verifies that the different chemical potentials of the graphene can make the graphene-based devices/systems achieve the reconfigurable frequency property.

**Figure 7:**
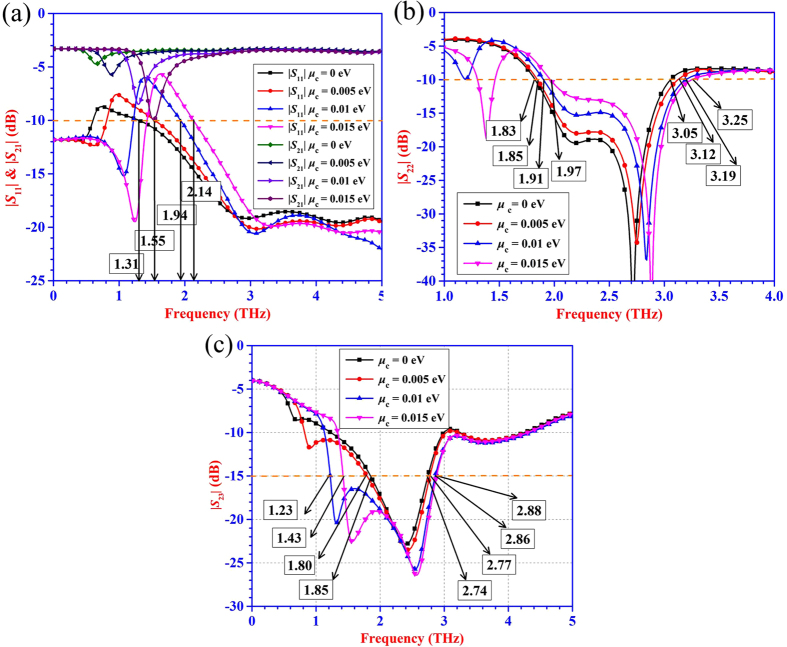
The simulated scattering parameters of graphene-based power divider using uniplanar coplanar waveguide transmission line. (**a**) The curves of |*S*_11_| and |*S*_21_| with the changing chemical potentials. (**b**) The results of |*S*_22_ | when the graphene is loaded with different chemical potentials. (**c**) The curves of |*S*_23_| representing the variable port isolation with the chemical potentials changing from 0 eV to 0.015 eV.

The lossy transmission-line model and electrical parameter-extraction theory can be also applied to three-port devices. According to similar uniplanar power divider circuit structures^25^, a power divider utilizing graphene-metal uniplanar coplanar waveguide (strip) transmission line is proposed. Figure 3 shows the three-dimensional views of the proposed graphene-based uniplanar power divider. The corresponding scattering parameters are presented in Fig. 7(a–c). The power divider can achieve bandwidth reconfigurable function due to the introduction of the graphene with tunable chemical potentials. When |*S*_21_| is near to −3 dB, the energy can be divided equally into two output ports with extremely low insertion loss level. From Fig. 7(a), the initial frequencies where the magnitude |*S*_21_| is close to −3 dB and |S_11_| below −10 dB are 1.31 THz, 1.55 THz, 1.94 THz and 2.14 THz with the chemical potential changing from 0 eV to 0.015 eV. The initial frequencies increase as the chemical potential increasing. The tunable bandwidth function can be observed visibly from Fig. 7(b–c). The operating frequencies which |*S*_22_| below −10 dB move to higher frequency bands with the increasing chemical potential. The factor that greatly restricts the bandwidth of the proposed power divider is the isolation (|*S*_23_|) between two output ports. In order to make the proposed graphene-based power divider have reliable performance, |*S*_23_| should be below −15 dB. Thus the function of tunable bandwidth can be achieved when it meets the condition of |*S*_22_| < −10 dB, |*S*_23_| < −15 dB, |*S*_11_| < −10 dB and |*S*_21_| ≈ −3 dB. The detailed information is also tabulated in the Table 1.

## Discussion

The frequency reconfigurable graphene-metal transmission line based on lossy transmission-line model and electrical parameter-extraction (T-LMEP-E) theory is proposed in this paper. Three kinds of transmission lines, including microstrip line, double-side parallel strip line and uniplanar coplanar waveguide (strip) are simulated to verify the proposed theory. The values of attenuation constant and phase constant, and the real and imaginary parts of the complex the characteristic impedance are extracted to analyze in details, respectively. When the attenuation constant and the reactance part of the characteristic impedance are both close to zero, the transmission line is under the transmission mode with extremely low insertion loss. While the attenuation and the reactance part of the characteristic impedance have considerable value, the transmission line is under the radiation mode with large attenuation. The phase constant, as a function of frequency, has a linear relationship under the transmission mode. Within the range of linear relationship, the values of phase constants are different when the graphene is loaded with dynamic chemical potentials at the same frequency, indicating the tunability of the operating frequency. Thus the operating frequency can be altered with changing chemical potential *μ*_c_. This theory can be applied for the typical three-port devices such as power dividers. The principle of the bandwidth reconfigurable uniplanar coplanar waveguide (strip) power divider is the same with that of transmission lines. Furthermore, this work will provide a potential approach and design theory to other kinds of microwave, terahertz and mid-infrared multi-port devices/systems requiring frequency reconfigurable properties.

## Additional Information

**How to cite this article**: Wu, Y. *et al*. A Generalized Lossy Transmission-Line Model for Tunable Graphene-Based Transmission Lines with Attenuation Phenomenon. *Sci. Rep*. **6**, 31760; doi: 10.1038/srep31760 (2016).

## Figures and Tables

**Table 1:** The impedance bandwidth of the graphene-metal uniplanar coplanar waveguide (strip) power divider.

Conditions	*μ*_c_ = 0 eV	*μ*_c_ = 0.005 eV	*μ*_c_ = 0.01 eV	*μ*_c_ = 0.015 eV
|*S*_22_| < −10 dB	1.22 THz (1.83 THz–3.05 THz)	1.27 THz (1.85 THz–3.12 THz)	1.28 THz (1.91 THz–3.19 THz)	1.28 THz (1.97 THz–3.25 THz)
|*S*_23_| < −15 dB	0.89 THz (1.85 THz–2.74 THz)	0.97 THz (1.8 THz–2.77 THz)	1.63 THz (1.23 THz–2.86 THz)	1.45 THz (1.43 THz–2.88 THz)
|*S*_22_| < −10 dB	0.89 THz (1.85 THz–2.74 THz)	0.92 THz (1.85 THz–2.77 THz)	0.92 THz (1.94 THz–2.86 THz)	0.74 THz (2.14 THz–2.88 THz)
|*S*_23_| < −15 dB				
|*S*_11_| < −10 dB				
&|*S*_21_| ≈ −3 dB				
